# Prehospital treatment of sepsis: what really makes the “golden hour” golden?

**DOI:** 10.1186/s13054-014-0697-4

**Published:** 2014-12-17

**Authors:** Sarah A Sterling, Michael A Puskarich, Alan E Jones

**Affiliations:** Department of Emergency Medicine, University of Mississippi Medical Center, 2500 N. State Street, Jackson, MS 39216 USA

## Abstract

The early recognition of severe sepsis is important; however, early identification of severe sepsis can be challenging, especially in the prehospital setting. As previous research has shown, advanced notification of time-sensitive disease states by prehospital personnel can improve outcomes and time to initiation of treatments. Prehospital personnel can potentially impact outcomes in sepsis through early identification and treatment implementations, improving processes of care and transition of care. Further research is needed for a full evaluation of prehospital treatment effects of identification of sepsis and treatment by prehospital personnel and the impact on outcomes.

## Commentary

In a previous issue of *Critical Care*, Seymour and colleagues [1] performed a prospective observational study on the effect of prehospital intravenous (IV) catheter placement and fluid resuscitation in patients with severe sepsis. The authors should be commended for their efforts, as prehospital research can be particularly challenging, often requiring great forethought and planning. It is obvious that they thoughtfully and carefully approached their study, in both data collection and statistical analysis.

Despite much research and effort directed at the improved recognition and treatment of sepsis, sepsis-related mortality remains high [2]. Although various miracle drugs and iterations of resuscitation protocols have come and gone [3-5], perhaps the most consistent finding is that early recognition of severe sepsis and septic shock improves outcomes [6,7]. Simple identification of a disease state may seem like an easily obtainable goal, but recognition of sepsis, and even septic shock, is challenging. If recognition of sepsis remains elusive for the intensivist and emergency medicine physician with laboratory and imaging adjuncts, it is that much more difficult for prehospital personnel who must rely on limited clinical indicators alone. Systemic inflammatory response criteria, though a somewhat useful initial screening tool, are poorly specific for sepsis [8]. Although previous authors have attempted to develop screening tools for prehospital identification of patients with severe sepsis, these tools have yet to be validated and sometimes require lactate measurement not available to most prehospital personnel [9].

Seymour and colleagues [1] suggest that, in patients with severe sepsis, prehospital fluid resuscitation is associated with improved mortality. If it is assumed that an oxygen supply and demand mismatch represent the primary pathology in sepsis and that prompt fluid administration helps to improve this, it is tempting to interpret these data to suggest that even earlier fluid administration to patients with sepsis leads to decreased mortality. Patients who received IV fluids or access were rated as more acutely ill in this study, and those who received IV fluids had a lower systolic blood pressure; so it is surprising that these patients, the sicker patients, would have better adjusted mortality rates. Or is it?

Interestingly, patients who received an IV catheter alone demonstrated improved adjusted mortality rates compared with those patients who received neither an IV catheter nor fluids, with adjusted odds ratios similar to those of patients who received IV fluids. In their discussion, the authors rightly note that placement of an IV catheter may be influencing processes of care. Perhaps placement of an IV or administration of IV fluids is simply a marker of severe sepsis recognition. As noted, advanced identification and notification by prehospital personnel are associated with improved outcomes and time to procedure in other conditions such as ST elevation myocardial infarction and stroke [10,11]. Similarly, previous research has shown that a written diagnosis of sepsis by prehospital personnel is associated with improved times to antibiotics and initiation of goal-directed therapy [12].

Of course, a provider cannot only recognize severe sepsis; they must use this diagnosis and promptly proceed with a treatment plan to ultimately affect outcomes. Another potential explanation for the difference in mortality is that patients arriving with IV access had fewer delays in the implementation of physician orders. Although obtaining IV access alone is typically not a time-consuming process, the cumulative delay of increased triage times and time to obtain IV access, start IV fluids, and initiate antibiotics could be quite significant.

In further support of the interpretation that processes, not simply prehospital IV fluids or access, drive the study results is the fluid volume administered. With a median fluid volume of 500 mL, it seems difficult to accept that this volume of fluid substantially altered mortality or organ failure score in and of itself. However, given the relatively low risk of complications associated with IV fluid resuscitation, especially the volume transfused during typical transport times, it seems like a relatively low-risk intervention, with definite potential benefits that further studies should explore.

In summary, we believe that the findings of Seymour and colleagues [1] are more likely a representation of improvements in downstream processes of care rather than fluid administration or IV access *per se*. As a result, we believe these data actually have much broader implications. The recognition of severe sepsis is crucial to the early diagnosis and management of sepsis, and the prehospital setting is no exception. Prehospital personnel can potentially play a vital role in improving outcomes by early identification and initiation of treatment, improving processes of care. While severe sepsis remains a unique and challenging disease state, optimal outcomes and treatment plans require a team approach incorporating nurses, physicians, and prehospital personnel, with education and training on the recognition and early identification of severe sepsis and septic shock.

## Abbreviation

IV: Intravenous
